# Cell Dispersal Influences Tumor Heterogeneity and Introduces a Bias in NGS Data Interpretation

**DOI:** 10.1038/s41598-017-07487-z

**Published:** 2017-08-04

**Authors:** Lőrinc Pongor, Hajnalka Harami-Papp, Előd Méhes, András Czirók, Balázs Győrffy

**Affiliations:** 1 MTA TTK Lendület Cancer Biomarker Research Group, Institute of Enzymology, Budapest, Hungary; 2 Semmelweis University 2nd Department of Pediatrics, Budapest, Hungary; 3 Department of Biological Physics, Eötvös Loránd University, Budapest, Hungary; 4 Department of Anatomy and Cell Biology, University of Kansas Medical Center, Kansas, USA

## Abstract

Short and long distance cell dispersal can have a marked effect on tumor structure, high cellular motility could lead to faster cell mixing and lower observable intratumor heterogeneity. Here we evaluated a model for cell mixing that investigates how short-range dispersal and cell turnover will account for mutational proportions. We show that cancer cells can penetrate neighboring and distinct areas in a matter of days. In next generation sequencing runs, higher proportions of a given cell line generated frequencies with higher precision, while mixtures with lower amounts of each cell line had lower precision manifesting in higher standard deviations. When multiple cell lines were co-cultured, cellular movement altered observed mutation frequency by up to 18.5%. We propose that some of the shared mutations detected at low allele frequencies represent highly motile clones that appear in multiple regions of a tumor owing to dispersion throughout the tumor. In brief, cell movement will lead to a significant technical (sampling) bias when using next generation sequencing to determine clonal composition. A possible solution to this drawback would be to radically decrease detection thresholds and increase coverage in NGS analyses.

## Introduction

Accumulated genetic changes in a malignant tumor comprise somatic mutations and copy number changes, gene expression alterations, and epigenetic modifications. The differential combination of these traits in individual cells leads to intratumor heterogeneity which helps a tumor to increase survival, acquire metastatic capabilities and to develop resistance against systemic chemo- and targeted therapies^1^. In other words, intratumor heterogeneity can be interpreted as an evolutionary process which leads to a continuously increasing number of distinct clones within the primary tumor mass^2^. The presence of these distinct genotypes can give fitness advantage to a particular tumor clone at a certain stage and is therefore a driving force of the malignant progression. Dispersal is a crucial factor in these evolutionary processes, however little is known about the role of cell dispersal and motility in tumorigenesis.

From the theoretical point of view, evolution of a tumor can be either linear when mutations follow each other in a serial order so that a specific lineage will contain all previous mutations^3^, or it can be branched i.e. lineages will contain a different sets of mutations^4^. Recent multi-region sequencing studies are in favor of the branched evolution model in tumors^5^. When the mutation profiles of cancer patients are examined, we can identify mutations present in each of the samples obtained from a single tumor. Other mutations are specific to few samples only. Interestingly, some of the patients show low genetic (mutational) heterogeneity, in which case almost all of the mutations are either common in all intra-tumor samples, or exclusive in the primary tumor. The underlying process that may cause these conserved mutational profiles is unknown – a possible explanation may be a sub-clone selection process caused by clonal competition^4^.

The Cancer Genome Atlas (TCGA) of the National Cancer Institute (http://cancergenome.nih.gov/) has published a large number of breast cancer samples investigated with NGS^6^. The results were in general agreement with the current dogma of clinical practice i.e. high mutation rates entail a lower survival rate. This established view was however questioned by findings that even marginal clones can have a prominent effect on the patients’ response to therapy and survival after drug treatment^7, 8^. Moreover, a careful statistical re-analysis of TCGA data showed that a slight change to the detection strategy can lead to a six-fold increase in the number of potentially relevant mutations^9^ which again points to the difficulty of relating mutational frequencies to prognosis.

**Figure 1:**
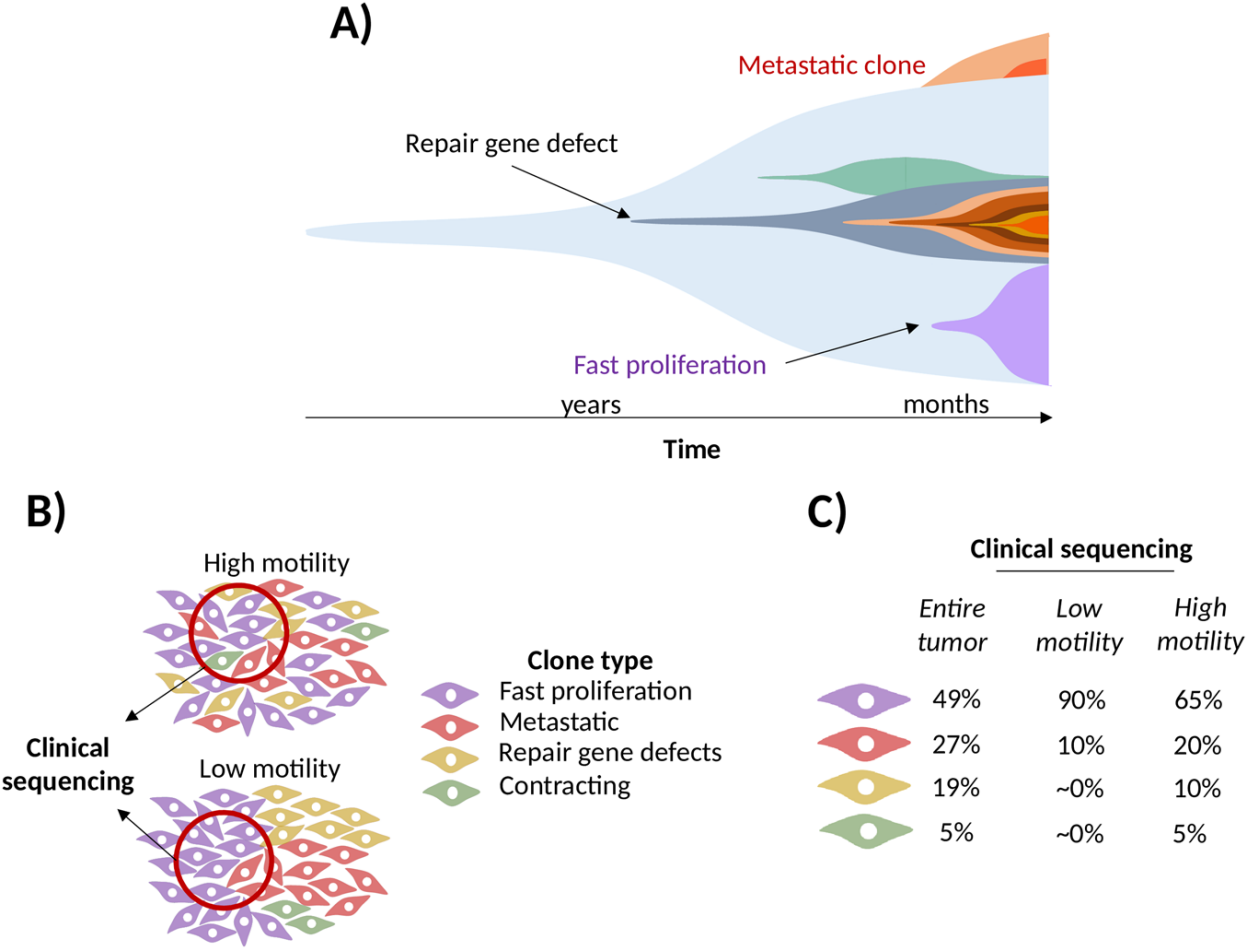
Theoretical opportunities of correlation between tumor composition and cell dispersal. During its course, the tumor accumulates multiple mutations (**A**). Sample collection in a model without motility will only acquire some of the clones present in the entire tumor (**B**). In contrast, high motility tumors will include multiple different clones in a single sample. These will impact on cellular composition when the sample is sequenced (**C**).

Recently, it was shown that colorectal cancer cell lines harboring different mutations in signal transduction pathways can have different migratory potential^10^. Features related to cytoskeletal mechanisms affect tumor growth and metastasis^11^, and altered pathways can influence multiple key genes involved in these processes^12, 13^. There is an important additional implication in case the mutations are detected by NGS: a mutation enabling rapid cell dispersal will result in lower number of these cells in a particular tumor sample (Fig. 1). By setting high detection cutoff values, faster moving clones will be either diluted to sub-clonal frequency levels, or will remain undetected, either way causing misinterpretation. Therefore, we cannot exclude that apparently less heterogeneous tumor samples that are generally considered less dangerous according the current dogma, can in fact have a much worse prognosis simply because their clinically relevant mutations and their high mutational frequency levels are diluted below sub-detection levels.

The basic assumption of the present work is that high cell motility will lead to faster cell mixing within tumors and thereby lower the chance of detecting intratumor heterogeneity. Thus, depending on mutation composition, apparently homogeneous tumors can have actually worse prognosis than heterogeneous ones. In order to appraise these issues, we evaluated a model for cell mixing that investigates how short-range dispersal and cell turnover can account for cell mixing inside a tumor. We then utilized next generation sequencing in this model system to establish optimal detection cutoff values capable to capture motility-related mixing. Finally, we assessed the effect of motility-related mutations on clinical outcome in a sizeable set of patient samples. We found that increased cell motility is in fact likely to cause a bias in detecting tumor heterogeneity by NGS analysis, an issue that may need to be dealt with in the clinical practice.

## Materials and Methods

### Cell culture

A total of four human cell lines deriving from melanoma were used in the study. The A375 GFP cell line (LINTERNA A375 GFP tagged expresses green fluorescent protein as a free cytoplasmic protein, it possesses bright green fluorescence at 482/502 nm excitation/emission wave) was purchased from Innoprot. The Mel-Juso RFP cell line (Mel-Juso-TurboFP602-GVO-CD) was purchased from BioCat. SK-MEL-28 cell line was purchased from CLS Cell Lines Service and the fourth cell line was our previously used MeWo^14^.

The Mel-Juso cell line was maintained in RPMI-1640 medium, the A375 GFP, SK-MEL-28 and MeWo cell lines were cultured in DMEM medium (Lonza, Switzerland; with 4500 mg/dm3 glucose, piruvate and L-glutamine), each supplemented with 10% fetal calf serum (Lonza, Switzerland) and 1% penicillin–streptomycin–amphoterycin (Lonza, Switzerland) in tissue culture flasks at 37 °C in a humidified 5% CO_2_ atmosphere.

### DNA isolation and quality control

Genomic DNA was extracted directly from each cultured cell line for mutation validation. After trypsin-EDTA treatment for disruption of cell monolayers the genomic DNA was extracted from the cell suspension with DNeasy Blood and Tissue Kit (Qiagen, Germany) according to the manufacturer’s recommendations. Approximately 500 ng DNA was extracted from ~5 × 10^5^ cultured cells for each PCR reaction. Genomic DNA was also extracted from the samples of the Ring cell invasion assay (*see Ring cell invasion assay section*). In this, the sampling was performed by cell scraper (VWR, Hungary), the scratched cells were raised in growth medium, and the genomic DNA was extracted directly from the cell suspension. In each case, the concentration (ng/µl) and purity (absorbance at 280 and 260 nm) of the DNA was measured by a Nanodrop ND1000 spectrophotometer.

### Validation of cell line specific genotype

Presence of cell line specific homozygous and heterozygous mutations in the TP53, PTCH1, CDKN2A, BRAF, HRAS, NRAS, ZNF214 and NF1 were validated using Sanger sequencing. These mutations are also used to differentiate and to calculate the composition of cell line mixtures. The direct DNA sequencing was performed using genomic DNA amplified by polymerase chain reaction. Primers were designed to be located in flanking sequences previously described to contain a specific mutation in the gene according to COSMIC (cancer.sanger.ac.uk/cell_lines) to allow amplification of genomic DNA only. The used primers and primer features are listed in Supplemental Table 1.

The PCR reaction was performed in 25 µl final volume, containing 500 ng of genomic DNA, 10 mM of each dNTP (Invitrogen, CA, USA), 10 µM of each of the eight primers, 5 units of Taq polymerase (Invitrogen), 2.5 µl 10x buffer, completing to the final volume with nuclease free H_2_O. The amplification reaction was carried out in a thermocycler (Swift Maxi, ESCO) with an initial denaturation step of 3 min at 94 °C, followed by 35 cycles consisting of three steps: 94 °C for 30 sec, 53 °C for 30 sec, and 72 °C for 2 min. Annealing temperature was optimized to the primers melting temperature. The last cycle was followed by an extension step of 6 min at 72 °C. The PCR product was purified, and DNA sequencing was performed at the Department of Genomic Medicine and Rare Disorders (Semmelweis University, Budapest, Hungary). The DNA sequence was analyzed by BioEdit and Genedoc programs.

### Ring cell invasion experiment

FlexiPERM^®^ conB cell exclusion silicone rings were purchased from Sarstedt AG&Co. (Nümbrecht, Germany), and were preserved in pure alcohol solution until use. The rings were placed vertically in 60 × 15 mm culture dishes (Sarstedt AG&Co., Germany). In this setting, the inner cultivation area was 3.1 cm^2^ per unit and the external growth surface was 17.9 cm^2^/well. Outline of the rings were marked on the transparent plate bottom. The first cell suspension was added carefully to the inner side of the ring with a cell number of 300,000 cells inside the ring in 3 ml volume of DMEM medium. After an overnight incubation for cell attachment, the rings were carefully removed using tweezers followed by washing the cells with 1x phosphate-buffered saline (PBS) to remove cell debris. The second cell line (MeWo in each case) was dispensed in the entire well surface in 6 ml of DMEM medium, with a concentration of 100,000 cells/ml. The marked borders of the silicone rings were monitored under light microscope (Leica Microsystems, Wetzlar, Germany). Growth medium was replenished every second day. Fluorescent photographs were taken at multiple sites along the external and inner border of the marked ring on the 7^th^ day (after 6 day of co-cultivation) (Leica Microsystems, Wetzlar, Germany) at the HAS Research Center for Natural Sciences. Images were processed by Image J software (National Institutes of Health, Bethesda, MD).

Sampling from the *Ring cell invasion experiment* was processed in two circular arcs within the cell culture dishes. The inner arc was positioned directly next to the marked border of the formal ring, its width was 10 mm (inner sample), and the second arc was positioned directly to the wall of the dish, its width was 10 mm (external sample). Sampling was performed by a sterile cell scraper. The cell combination sample set was contained 2/5/10/25 or 50% LINTERNA^TM^-A375 cells among MeWo cells in final 1 × 10^6^ cell/ml concentration.

We computed Wilcoxon signed rank test to compare mutation frequencies between invading cell lines and MeWo, and to compare the degree of invasion between A375 and SK-MEL-28.

### Video microscopy

Time-lapse recordings were performed on a computer-controlled Leica DM IRB inverted microscope equipped with a Marzhauser SCAN-IM powered stage and a 10x N-PLAN objective with 0.25 numerical aperture and 5.8 mm working distance. The microscope was coupled to a Zeiss Colibri illumination system and an Olympus DP70 color CCD camera for epifluorescent image acquisition.

Cell cultures were kept at 37 °C in humidified 5% CO_2_ atmosphere in tissue culture grade Petri dishes (Greiner, Germany) in a mini incubator (CellMovie) mounted on the powered stage of the microscope. A custom made time-lapse experiment manager software controlled the field of view and plane positioning, illumination and image acquisition on a PC. Phase contrast and epifluorescent images of cells were collected consecutively every 10 minutes from each of the microscopic fields for up to 72 hours. Images were edited using NIH ImageJ software.

### Quantification of cell velocity and motility

Cell motility was quantified as the net displacement of tracked cells during the first 24–72 h of the recorded time period. The velocity, *v*_*i*_(*t*), of a given cell *i* at time *t* was calculated as *v*_*i*_(*t*) = *|x*_*i*_(*t* + Δ*t*) − *x*_*i*_(*t*)*|*/Δ*t* with a suitably chosen Δ*t*. We selected Δ*t* = *1* *h*, where the typical cell displacements are larger than *10* μm, hence larger than the error of the manual tracking procedure performed with the help of a custom-made cell tracking software.

Average velocity given by *F*(*v*) were calculated as shown in eq. (1):

1 $$v(t)=1/N(t){\sum }_{i=1}^{N(t)}{v}_{i}(t)$$

where the summation goes over each *N*(*t*) cell being in the cell population. The velocity distribution function *F*(*v*) gives the probability that for a randomly chosen *i* and *t* the velocity *v*_*i*_(*t*) is larger than *v*. Average distance of cell migration, *d*(*τ*), was calculated for a range of elapsed time lengths *τ* as: *d*(*τ*) = 〈|*x*_*i*_(*t* + *τ*) − *x*_*i*_(*t*)|〉_*i*,*t*_ where the average 〈…〉_*i*,*t*_ is taken over each possible choice of *t* and *i*.

### Ion Torrent Sequencing

Amplicons were designed using the AmpliSeq Designer software (Life Technologies, CA, USA), targeting the entire coding sequence of the genes. Primers were designed to include parts of the introns to achieve a higher coverage of the coding exons. Amplicon library was prepared with the Ion AmpliSeq Library Kit 2.0. In this, primer pools were added to 10 ng of genomic DNA and PCR amplified. PCR cycles were set up to include 18 cycles of 99 °C for 2 min, at 99 °C for 15 s, and at 60 °C for 4 min, and finally a plateau at 10 °C. Primers were partially digested using a FuPa reagent, and then sequencing adapters were ligated to the amplicons. Library was purified using the Agencourt AMPure XP Reagent (Beckmann Coulter, CA, USA). The final library concentration was determined by fluorescent measurement on Qubit 2.0 instrument (Life Technologies, CA, USA). Template preparation was executed using an Ion OneTouch kit on semiautomated Ion OneTouch instrument using the emPCR method. After breaking the emulsion, nontemplated beads were removed from the solution during the semiautomated enrichment process on Ion OneTouch ES instrument. Following adding the sequencing primer and polymerase, the fully prepared Ion Sphere Particle (ISP) beads were loaded into an Ion 314 sequencing chip, and the sequencing runs were performed using the Ion PGM 200 Sequencing kit (Life Technologies, CA, USA). Average sequencing coverage was 600x (range 200–1200x).

The sequencing was run using three regions/well with one repeat for each of the three cell lines (3 × 3 × 2 = 18 measurements). The dilution cascade was measured in duplicates (2 × 5 = 10 measurements). Four additional repeats were added as technical controls (total number of sequencing samples n = 32).

### Data Analysis

Data from the Ion Torrent runs were analyzed using the platform-specific pipeline software Torrent Suite v3.2.1. Quality control of reads was performed with the *FastQC*, followed by read trimming using *trimmomatic* ^15^. High quality reads where mapped to the human genome (GRCh37, Ensembl) using *BWA MEM* ^16^, and converted to *bam* format using *SAMtools*. Aligned reads where processed using the *GATK* toolkit^17^ based on the GATK pre-processing best practices (https://www.broadinstitute.org/gatk/guide/bp_step.php?p=1). Mutation calling was performed using the SAMtools mpileup (http://www.htslib.org/) default parameters. Mutations were identified by first selecting regions with cell line specific mutations from the SAMtools mpileup output, followed by calculation of reference and non-reference base totals at all positions. In case of non-reference, bases had to match the alteration of the cell lines.

### Modeling of cell dispersal and tumor heterogeneity

In addition to the experimental investigations, we set up a computational model in order to correlate the experimentally observed cell behavior with a simple mathematical description of cell dispersal and heterogeneity.

This model uses an *N x M* sized matrix to simulate the surface of a plate. Each *x*, *y* coordinate pair represents a position on the plane where a cell can be placed, and movement outside of the boundaries of the matrix is prohibited. The model contains a few simplifying assumptions: cells cannot overlap, environment (e.g. medium concentration through time) is constant, apoptosis is determined as a random function, and the size of cells is identical.

Two cell types are used in each simulation, agreeing with the experiments of the invasion assay. The first cell line (inner) is placed in the middle of the plate simulating a silicone ring with a radius N × 0.1 (radius is 10% of height), while the second cell line was evenly distributed across the entire surface. The density of the inner cell line was 5%, while density of the second cell line was set to 2.5%.

Cell velocity characteristics measured in the invasion assay were used during simulation. Each cell type “ ***i***” had a clonal persistence (velocity) ***C***_***i***,***clone_persistance***_ of 5, 13 and 15 for the MEWO, SKMEL28 and A375 respectively, where each simulated cell “ ***j*** “ had persistence randomly selected using eq. (2):

2 $${C}_{j,icellpersistance}=rand(2\ast {C}_{i,clonepersistance})$$

Persistence in the simulation is represented by a separate movement counter for each cell. Movement direction for each cell is randomly selected at each hour of the simulation. Clone division time *T*_*i*,*clone_division_time*_ was set to 22 h, 22 h and 19 h for the MEWO, SKMEL28 and A375 respectively, where division time for each simulated cell “ ***j*** ’’ was selected using eq. (3):

3 $${T}_{j,icellcelldivisiontime}=rand(2\ast {T}_{i,clonedivisiontime})$$

Division time for each cell was stored in a separate division timer showing the number of cycles needed to pass before division. The division timer decreases at each cycle, while the movement counter increases until the maximal travel distance is reached.

In the simulation 30 cycles represent one hour, the total time of the simulation is 6 days. During each cycle of the simulation, the first iteration through cells identifies candidates that will divide by decreasing the division timer until 0 is reached. Cell division involves creation of a daughter cell adjacent to the parent, where cell characteristics are randomly generated based on the previous sections (eqs 1 and 2). The second iteration identifies cells that are moving based on the persistence (movement counter). In case a cell moves, the cell is placed in a position where no other cell is present in a predetermined direction. In case a cell reaches the boundary of the simulation space or no space is available in the original movement direction, the direction is altered by inverting the *x* or *y* directions.

The modelling program was written in C programming language; data visualization was performed using the Allegro package version 5.2 (http://liballeg.org/). The program is available upon request.

## Results

### Cell dispersal and tumor velocity

**Figure 2:**
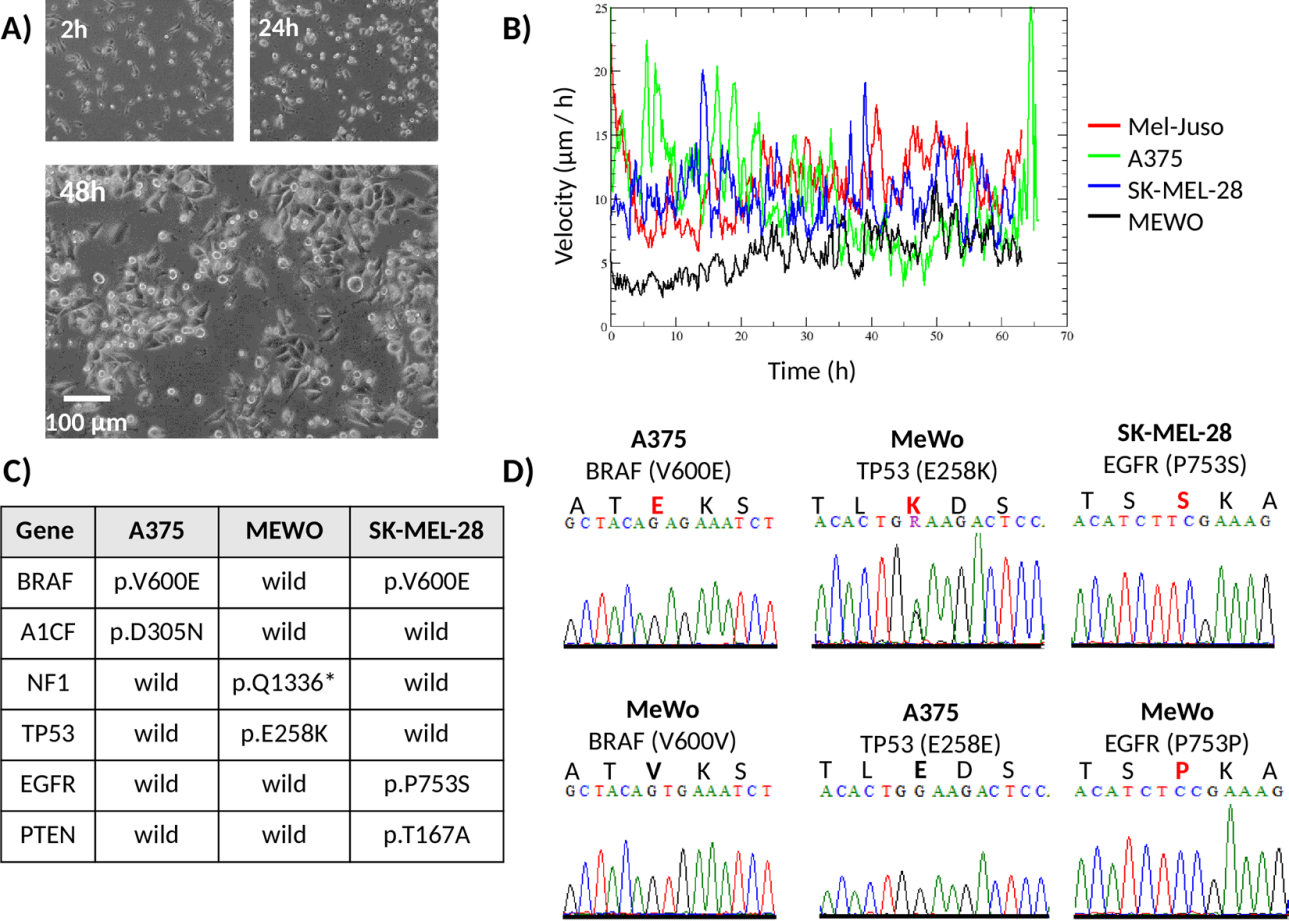
Migration of different cell lines. Video microscopy images of the highly active A375 (**A**). One can compute the absolute movement as a function of time and use this to compute cell line velocities (**B**). Selected key mutations for NGS sequencing (**C**) and mutations validated using Sanger sequencing (**D**).

By tracking the cell movement of each cell line separately by video microscopy (Fig. 2A), we were able to quantify cell motility, dispersal and velocities as well as movement directions. Cell displacement in monocultures showed random movement directions, while co-cultured cell displacement direction was mostly influenced by cell density, in which cells tend to move into lower density regions. The MEWO cell line had the lowest velocity (5 micron/h), while the other cell lines had similar high velocities around 12–13 micron/h (Fig. 2B). For this reason, we decided to perform ring cell invasion by pairing the two faster A375 and SK-MEL-28 cell lines with the slower MEWO cell line. Mono-culture video microscopy results can be found in the supplementary video material (Supplemental Video 1). The microscopy analysis results are displayed in Supplemental Fig. 1 comprising of cell line velocity (Supplemental Fig. 1A), displacement (Supplemental Fig. 1B) and velocity probabilities (Supplemental Fig. 1C).

To differentiate cell lines, and to calculate clonal compositions, a total of 6 key cell line specific mutations were selected in the BRAF, A1CF, NF1, TP53, EGFR and PTEN genes (Fig. 2C). A complete list of sequenced mutations can be found in the Supplemental Table 2. Three key mutations in the BRAF, TP53, and EGFR genes were further validated using Sanger sequencing (Fig. 2D).

### Ring cell invasion experiment proves marked infiltration and a reduction in homozygous mutation frequencies

**Figure 3:**
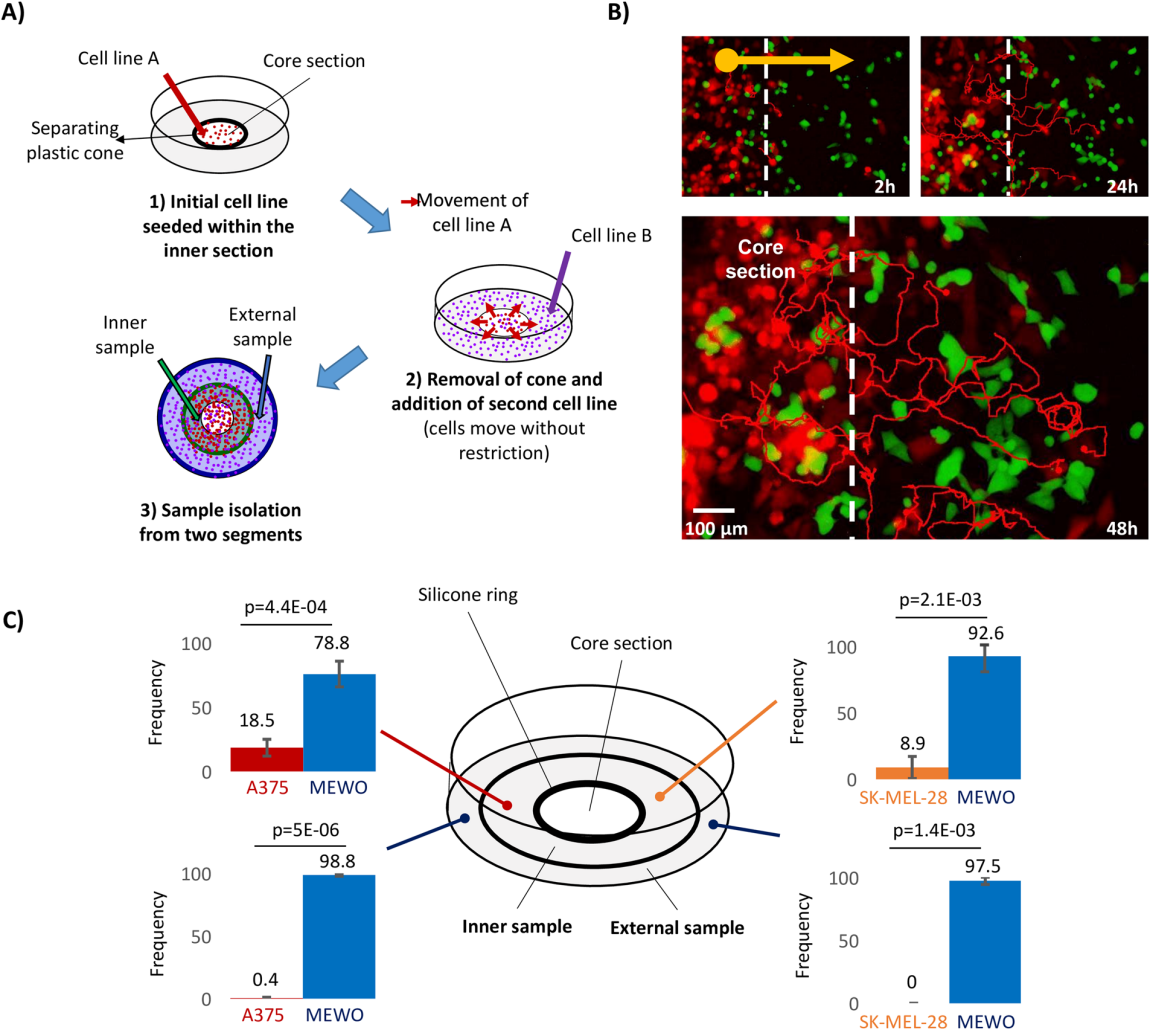
Ring cell invasion assay and migration trajectory. An experimental model of cell line dispersal utilizing three cell lines with dissimilar movement features. During the experiment, spatially separated differentially fluorescent cell lines are mixed in a two-step process (**A**). As a result, mixing of the two cell lines can be documented using fluorescent microscopy as displayed for A375 and Mel-Juso (**B**). Cellular composition measured using next-generation sequencing (**C**). In each case, the cell lines with higher velocities (A375: red and SK-MEL-28: orange) were paired with the MEWO (blue) cell line.

Cell dispersal and mixing was modelled using a ring cell invasion experiments (Fig. 3A). In these experiments, one cell line was placed in an inner circle (separated by a silicone ring) of a culture dish. After incubation and cell adhesion to the dish, the silicone ring was removed, and the second cell line (MEWO in each case) was placed on the entire surface of the dish. Invasion is represented as cells leaving the inner core area towards the external rings. Cell invasion was measured by following cells using fluorescent video microscopy (Fig. 3B). Video microscopy results of cell dispersal using the A375 and Mel-Juso fluorescent cell lines can be found in Supplemental Video 2. During each experiment, two samples were isolated and used in the NGS decomposition analysis. The first sample was collected with a 10 mm radius surrounding the perimeter of the silicone ring, while the second sample was collected from the external region of the culture dish (Fig. 3A).

Sequencing results of the A375-MEWO pairs show an infiltration rate of 18.5% in the internal sample with a proportion below 1% of A375 cells in the external region (Fig. 3C). Heterozygous mutation frequencies of the MEWO cell line were 42.0% in the inner region, and ~54.2% in the external region, which was in the expected range (Supplemental Table 3). Invasion sequencing frequencies). Interestingly, we identified a total of 45 reads that derived from the A375 cell line in the external region, with a total frequency of 0.5%. In case of the SK-MEL-28 and MEWO invasion assay, we observed an 8.9% infiltration rate in the inner sample, with no detectable SK-MEL-28 mutations in the external region (Fig. 3C).

Notably, when looking at the mean mutation frequencies of the dominant cell line in the ring invasion experiments, we see that even though the external samples of the SK-MEL-28 and MEWO pairs had no detectable infiltration, homozygous mutation frequencies already dropped to 97.5%. Interestingly, standard deviations of mutation frequencies detected in one sample ranged between 6–12% in case of the inner samples (Supplemental Table 3). Invasion sequencing frequencies), which may have significant effect on data interpretation. Mean frequency difference for mutation pairs in biological replicates was at 3.8% for high coverage mutations (range between 0–9%, standard deviation of 2.5%), and 7.6% including mutations with very low coverage (range between 0–34%, standard deviation of 9.6%).

The differences of mutation frequencies of the invading cell lines (A375 or SKMEL28) compared to MEWO were significant in all four settings (Fig. 3C). Mutation frequencies detected in the inner sections of the two invading cell lines were significantly different, where A375 had a higher degree of invasion compared to SKMEL28 (p = 0.011). Notably, when comparing homozygous mutation frequencies of the MEWO cell lines in the two invasion experiments, the difference in the degree of invasion was significant (p = 0.0059), which was not detectable (p = 0.39) when performing the analysis using heterozygous mutations. The difference in the external sample was not significant.

### Calibration runs uncover higher deviations for cells having lower proportions

**Figure 4:**
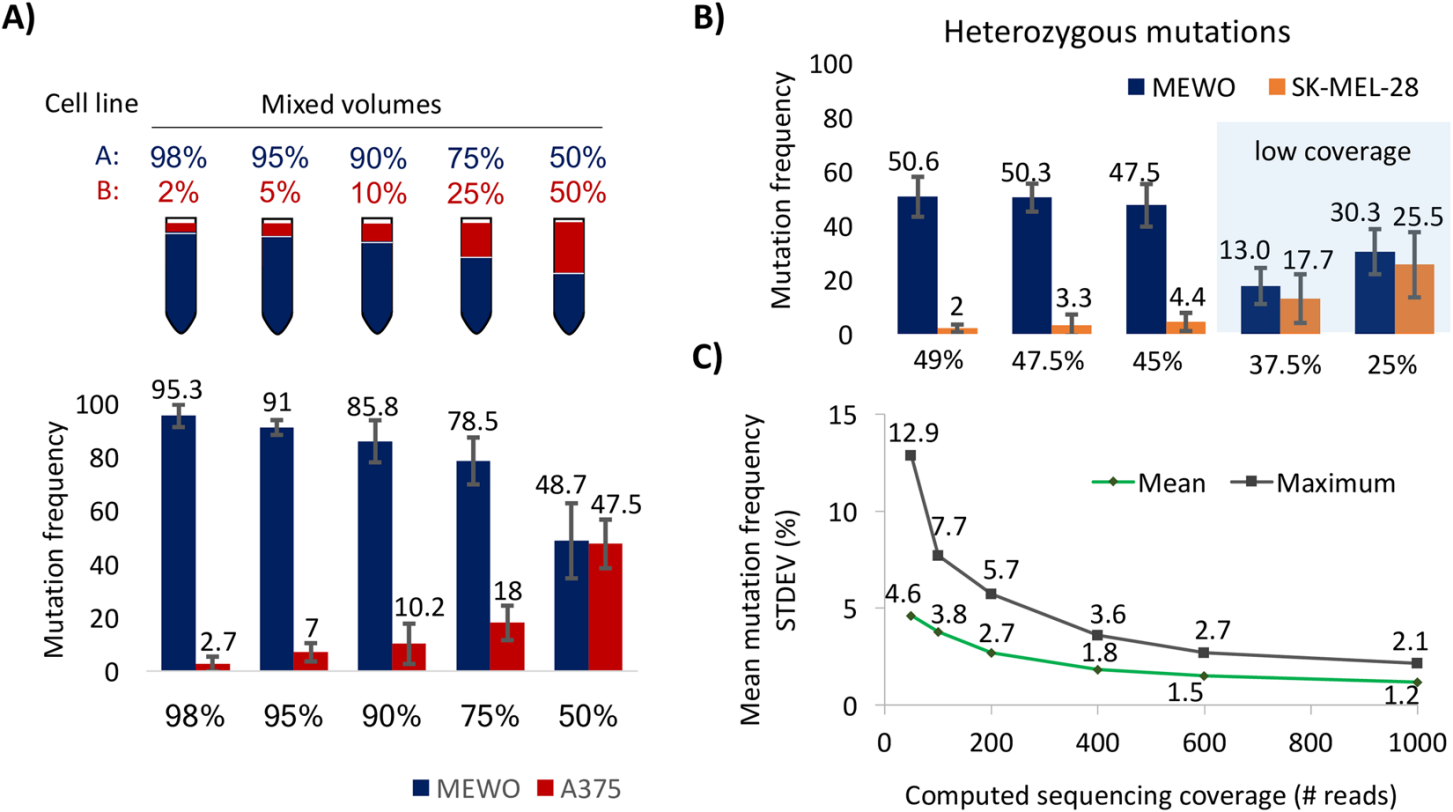
Correlation between cellular composition and mutation frequencies using next-generation sequencing. Calibration sequencing frequencies of the A375 and MEWO cell lines using homozygous mutations (**A**). Calibration sequencing frequency results of the SK-MEL-28 and MEWO pairs calculated from heterozygous mutations (**B**). Mean (green) and maximal (grey) mutation frequency standard deviations obtained from permutation test based on different coverages (**C**).

To better understand the linkage between cellular composition and mutation frequencies, we performed a calibration sequencing, where pre-determined volumes of two cell lines were mixed. The selected proportions were 2%, 5%, 10%, 25% and 50% of the A375 and MEWO cell lines as well as SK-MEL-28 and MEWO cell line pairs (Fig. 4A), each with technical replicates.

Higher proportion of one cell line generated frequencies with higher precision, while mixtures with even amounts of each cell line had lower precision manifesting in higher standard deviations ranging between 8–17% in case of high coverage. When including low coverage samples, standard deviation range increased to 30% (Fig. 4B).

Mean frequency difference for mutation pairs in technical replicates was 5.6% (mean ranging between 2.9–10.4%) in the case of the A375-MEWO pair. Similar results were obtained by the SK-MEL-28 and MEWO calibration sequencing technical replicates, where the mean frequency difference of mutations is 3.2% ranging between 1.6% and 5.6%. Interestingly, sequencing of the NF1 and PTCH1 genes in the A375 and MEWO pairings generated higher deviations, probably due to low coverage in one of the replicates at each combination.

Sequencing coverage in the calibration sequencing and ring invasion assays spanned between 100x–1200x. Coverage had a great effect on mutation frequencies in our experiments, which we further examined using a permutation test. Permutation test to calculate mutation frequency standard deviations was performed by generating/simulating reads with a predetermined mutation frequency, followed by shuffling of reads and calculation of the “measured” mutation frequency with different coverages. This process was done in five steps: 1) generating an array of 10000 elements (representing reads), 2) mutating a given F(expected) percentage of the simulated reads for frequencies between 1–99% separately, each percentage with 100 replicates, 3) shuffling of reads with Fisher-Yates shuffle, and 4) calculating the F(calculated) frequency of the first N reads representing total coverages of 50x, 100x, 200x, 250x, 400x, 500x, 600x, 700x and 1000x, and finally 5) calculating the standard deviation between F(expected) and F(calculated) frequencies among replicates. In this, low read coverage of a mutation generated higher average and maximum standard deviations (4.6% and 12.9% respectively), which decreased to less than 3% as sequencing coverage increased (Fig. 4C).

### Establishing an *in silico* model algorithm

**Figure 5:**
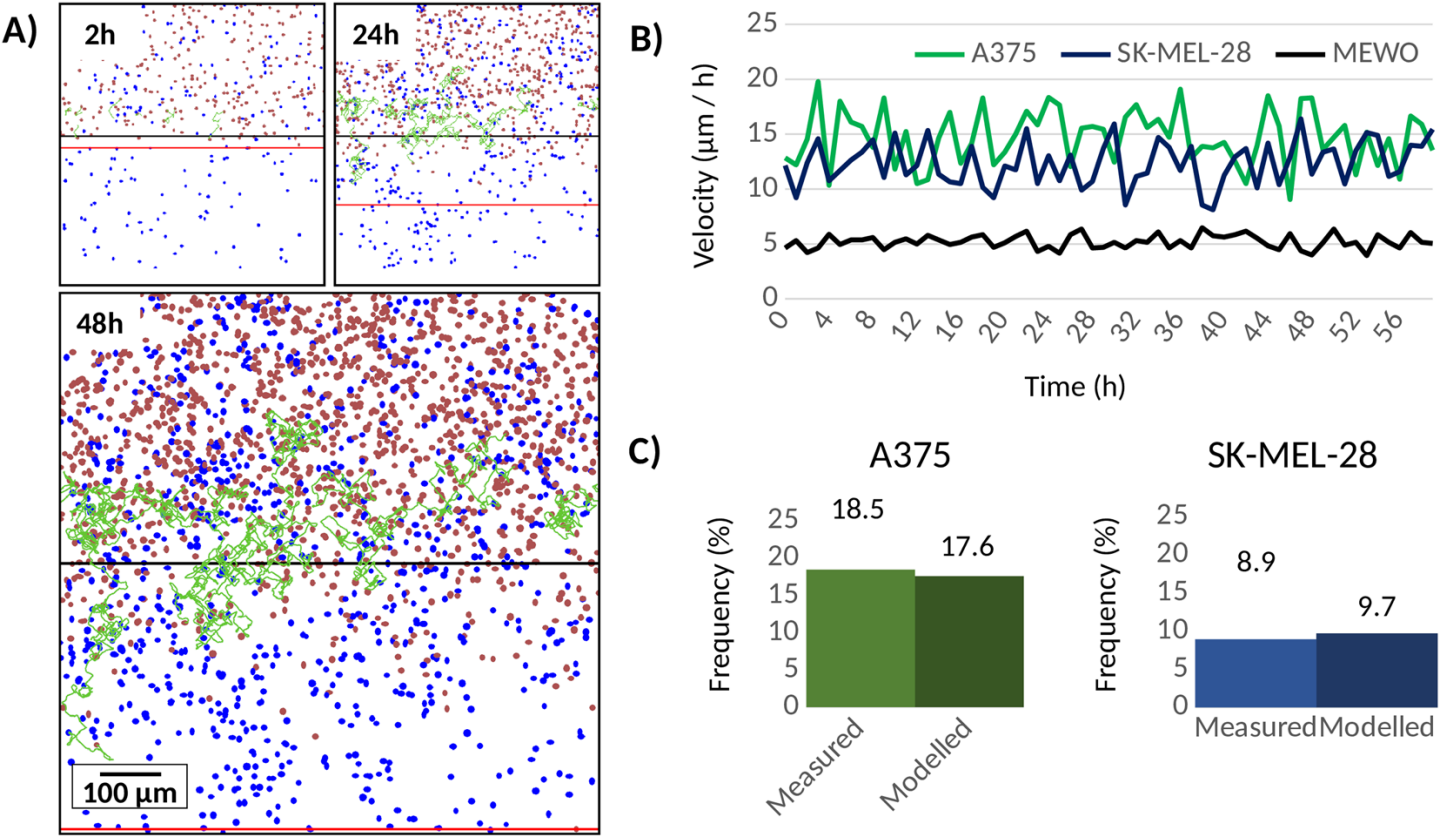
An *in silico* modelling of cell mixing. Visualization of the *in silico* model of cellular dispersal with maximal cellular distance marked by horizontal red line (**A**). Modeled cell velocities show stable cell speed during the simulation process for all four cell lines (**B**).

*In silico* modelling was performed utilizing phenotype data obtained from the mono-culture experiments. Approximate cell doubling time was set 22 hours for the MEWO, 19 hours for the A375 and 22 hours for the SK-MEL-28 cell line. Cell movement was set using data measured at 10-hour movement: MEWO cell line movement was set to 5 microns, while the other cell lines were set to 12 microns. In line with the monoculture experiments, simulations lasted for a total of 72 hours. By tracking the movement of a few cells, we generated a random movement pattern analogous to the cell culture experiments (Fig. 5A), Using the simulated data, we computed cell velocities which resembled the experimental results (Fig. 5B). The software can be downloaded as Supplemental Material 1.

Using the set parameters, we modelled cell mixing between the A375 and MEWO, and SK-MEL-28 and MEWO cell line pairs. The obtained mixtures of the simulated inner and external samples were similar to compositions measured during the Ring cell invasion assay. The simulated progression in this setup identical to the Ring cell invasion experiment is presented in Supplemental Video 4.

## Discussion

Clinical diagnostic sequencing of tumors usually involves analysis of genomic alterations using biopsies, with special attention to have a high proportion of tumor cells in the sample. Genetic composition of multiple regions in one tumor can be significantly diverse and even mutations practically undetectable at diagnosis can have a strong impact on survival and response to treatment^18, 19^. The identification of clinically relevant alterations is a puzzling task as less common mutations in heterogeneous tumors can be diluted below detection thresholds. In our experiments we show that cells can penetrate neighboring and distinct areas in a matter of days. Cellular frequencies measured will depend on the localization of sample acquisition and the extent of the samples.

Determining clonal and sub-clonal heterogeneity is usually accomplished by linking mutation counts, mutation frequencies, and copy number variation status^20^. Here, we found that sequencing predetermined mixtures of two cell lines generated high standard deviations in mutation frequencies ranging up to 17%, while the difference in technical replicates typically remained below 10% with a few exceptions. In a previous study, even using a high stringency of quality check metrics, the proportion of erroneous variant calling stretched over 20% in a single experiment – although using an older sequencing platform^21^. These observations show that even though NGS sequencing is precise, a minor change in sample preparation and analysis (e.g. issues connected to sample acquisition, handling, and processing) can have dramatic results on the final genetic composition.

We have to note that the two distinct cell lines (A375 and SK-MEL-28) displayed different degree of invasion. When detection thresholds are set to an adequately sensitive level and the coverage is sufficiently robust, multi-region sequencing can enable to track such distinct clones and their contribution to intratumor heterogeneity in a clinical sample. Highly mobile subclones can be either less or more present in a given sample than less mobile subclones.

Interestingly, when sequencing pre-determined mixtures of two different cell lines, 50–50% distribution resulted in the highest standard deviation in the detected cellular proportions. This was especially prominent in case heterozygous mutations were evaluated. In addition, even with a coverage at 1000x the standard deviation of the mean mutation frequencies still remained as high as 2%. We have to emphasize that here we used only two cell lines in one experiment in each setting. In a patient-derived tumor sample the different clones can all together represent a minority compared to normal DNA originating from immune cells and the tumor environment. In turn, this will result in an even smaller proportion of actual tumor cells with a given mutation in the sample. All together these results suggests that current next generation sequencing is not yet fully capable of exactly determining clonal composition and intratumoral evolution.

Motility can affect the mutation detection using next-generation sequencing and a shift in frequencies may misleadingly be interpreted as a change in clonal composition. Higher volume of a given cell line generated frequencies with higher precision, while mixtures with lower amounts of each cell line had lower precision manifesting in higher standard deviations. This is an alarming observation emphasizing a possible bias when using next generation sequencing to monitor and estimate tumor progression, since mathematical models that predict evolution paths^22^ often use mutation frequencies as key input information^23^.

Here we also present an *in silico* simulation model of cellular mixing. By only setting a few basic parameters (division rate, velocity, etc.) the model delivered a results comparable to the actual experimental observations. These results strongly support our hypothesis and enables future reverse engineering tumor compositions in order to decipher the process of tumor evolution.

A limitation of our approach is the confinement of the investigation to a 2D model. A recent *in silico* study has pointed out the effects of certain mutations on clonal composition using 3D models. In this, novel driver mutations decreased genetic diversity, while lack of driver mutations lead to a heterogeneous tumor^24^. Another limitation is the use of unobstructed space easily filled by the cells in our *in vitro* experiments – such conditions are only indirectly comparable to *in vivo* tumors. On the other hand, the growth of a tumor lasting for several years provides a similar opportunity for cancer cells to achieve a slow mixing. Finally, in our experiments we sequenced a panel of genes using targeted sequencing. In most cases, variant callers either identify germline mutations or somatic mutations in which case a normal/tumor pair is required. Because of the small size of the panel, varying coverage, and the fact that we knew where to expect the mutations, we decided on performing the analysis using a simple approach focusing on cell line specific mutations. Consequently, the applied method does not include statistical or background filtering, and cannot be employed to compute mutation calling rates in dissimilar experimental situations.

In summary, we demonstrate that cell movement can significantly influence tumor composition. Current methods for studying intratumoral evolution assume that genetic lesions are homogenous between regions and represent a shared clonal origin between tumor cells. These models do not account for the fact that some cells are more mobile than others. This can lead to mis-calling clones as absent or non-contributing to heterogeneity in multi-region sequencing studies of tumor evolution. We propose an alternative hypothesis that some of the shared mutations detected at low allele frequencies represent highly motile clones that appear in multiple regions of a tumor owing to dispersion throughout the tumor. This mixing of cells can lead to a sampling bias when using next generation sequencing to determine clonal composition. A possible solution to this drawback would be to radically decrease detection thresholds and increase coverage in NGS analyses.

## Electronic supplementary material

Supplementary video 1

Supplementary video 2

Supplementary video 3

Supplementary table 1

Supplementary table 2

Supplementary table 3

Supplementary methods and figure
