# Supplementary material for: Cell Dispersal Influences Tumor Heterogeneity and Introduces a Bias in NGS Data Interpretation
Source: Sci Rep. 2017 Aug 4;7:7358. doi: 10.1038/s41598-017-07487-z (PMC5544774; doi:10.1038/s41598-017-07487-z)
Supplement: Supplementary file 7 — Supplementary methods and figure [file 41598_2017_7487_MOESM7_ESM.pdf]

CELL DISPERSAL INFLUENCES TUMOR HETEROGENEITY AND INTRODUCES A BIAS IN NGS DATA INTERPRETATION

Lőrinc Pongor[1,2], Hajnalka Harami-Papp[1,2], Előd Méhes[3], András Czirók[3,4], Balázs Győrffy[1,2]

[1] MTA TTK Lendület Cancer Biomarker Research Group, Institute of Enzymology, Budapest, Hungary

[2] Semmelweis University 2nd Dept. of Pediatrics, Budapest, Hungary

[3] Department of Biological Physics, Eötvös Loránd University, Budapest, Hungary

[4] Department of Anatomy and Cell Biology, University of Kansas Medical Center, Kansas, USA

Supplementary methods

PCR reactions used for Sanger sequencing

The PCR reaction was performed in 25 µl final volume, containing 500 ng of genomic DNA, 10 mM of each dNTP (Invitrogen, CA, USA), 10 µM of each of the eight primers, 5 units of Taq polymerase (Invitrogen), 2.5 µl 10x buffer, completing to the final volume with nuclease free H2O. The amplification reaction was carried out in a thermocycler (Swift Maxi, ESCO) with an initial denaturation step of 3 min at 94°C, followed by 35 cycles consisting of three steps: 94°C for 30 sec, 53°C for 30 sec, and 72°C for 2 min. Annealing temperature was optimized to the primers melting temperature. The last cycle was followed by an extension step of 6 min at 72°C. The PCR product was purified, and DNA sequencing was performed at the Department of Genomic Medicine and Rare Disorders (Semmelweis University, Budapest, Hungary). The DNA sequence was analyzed by BioEdit and Genedoc programs.

Quantification of cell velocity and motility

Cell motility was quantified as the net displacement of tracked cells during the first 24-72 h of the recorded time period. The velocity, $v_i(t)$, of a given cell i at time t was calculated using equation (S1)

S1 $$v_i(t) = \frac{|x_i(t+\Delta t) - x_i(t)|}{\Delta t}$$

with a suitably chosen Δt. We selected $\Delta t=1h$, where the typical cell displacements are larger than *10* μm, hence larger than the error of the manual tracking procedure performed with the help of a custom-made cell tracking software.

Average velocity given by *F(v)* were calculated as shown in equation (S2):

S2 $$v(t) = \frac{1}{N(t) \sum_{i=1}^{N(t)} v_i(t)}$$

where the summation goes over each *N(t)* cell being in the cell population. The velocity distribution function *F(v)* gives the probability that for a randomly chosen *i* and *t* the velocity $v_i(t)$ is larger than *v*. Average distance of cell migration, *d(τ)*, was calculated for a range of elapsed time lengths τ as equation (S3):

S3 $$d(t) = \langle |x_i(t + \tau) - x_i(t)| \rangle_{i,t}$$

where the average $\langle ... \rangle_{i,t}$ is taken over each possible choice of *t* and *i*.

Ion Torrent sequencing

Amplicon library was prepared with the Ion AmpliSeq Library Kit 2.0. In this, primer pools were added to 10 ng of genomic DNA and PCR amplified. PCR cycles were set up to include 18 cycles of 99 °C for 2 min, at 99 °C for 15 s, and at 60 °C for 4 min, and finally a plateau at 10 °C. Primers were partially digested using a FuPa reagent, and then sequencing adapters were ligated to the amplicons. Library was purified using the Agencourt AMPure XP Reagent (Beckmann Coulter, CA, USA). The final library concentration was determined by fluorescent measurement on Qubit 2.0 instrument (Life Technologies, CA, USA). Template preparation was executed using an Ion OneTouch kit on semiautomated Ion OneTouch instrument using the emPCR method. After breaking the emulsion, nontemplated beads were removed from the solution during the semiautomated enrichment process on Ion OneTouch ES instrument. Following adding the sequencing primer and polymerase, the fully prepared Ion Sphere Particle 7 beads were loaded into an Ion 314 sequencing chip, and the sequencing runs were performed using the Ion PGM 200

Sequencing kit (Life Technologies, CA, USA). Average sequencing coverage was 600x (range 200-1200x).

Supplementary Figures

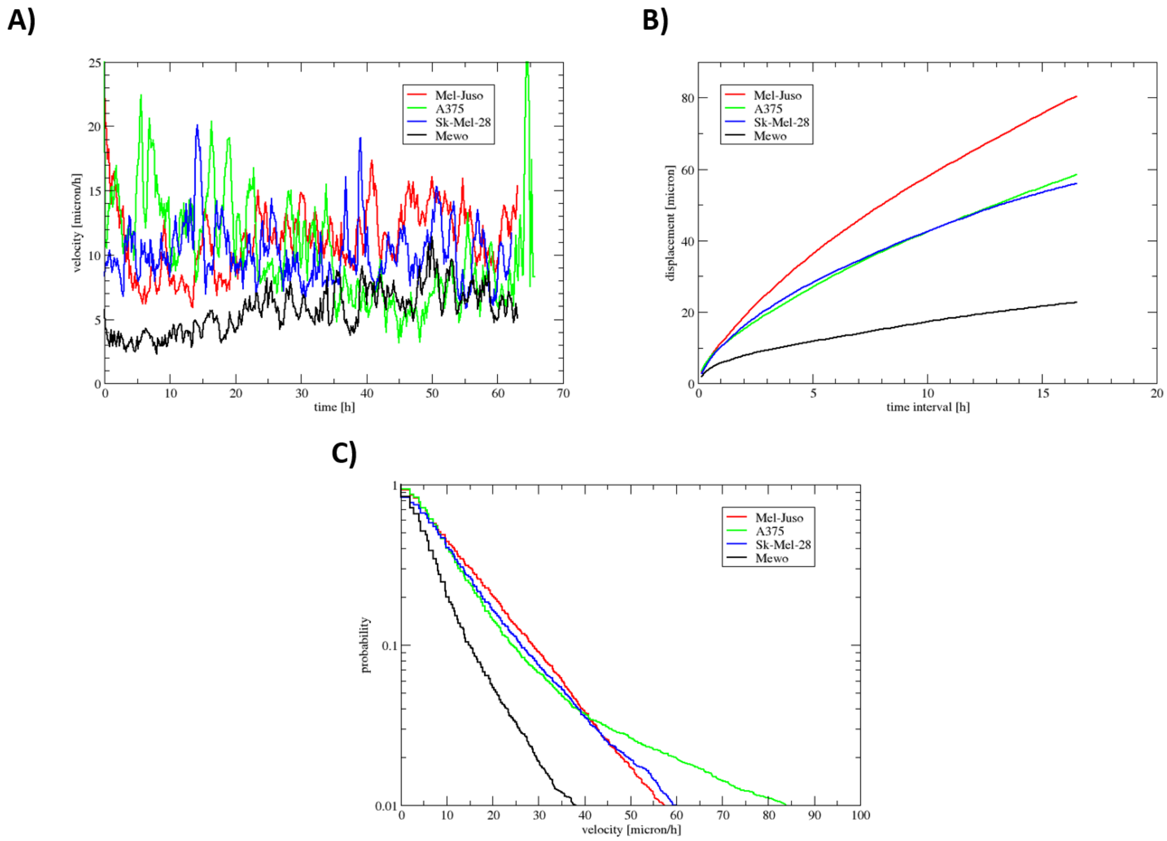

Supplemental Figure 1. Videomicroscopy results measured for each cell line showing velocity (A), displacement (B) and velocity probabilities (C).
